# Neurosurgery and the glymphatic system

**DOI:** 10.1007/s00701-024-06161-4

**Published:** 2024-06-21

**Authors:** Per Kristian Eide

**Affiliations:** 1 Department of Neurosurgery, Oslo University Hospital - Rikshospitalet, Nydalen, Pb 4950 N-0424 Norway; 2 Institute of Clinical Medicine, Faculty of Medicine, University of Oslo, Oslo, Norway; 3 KG Jebsen Centre for Brain Fluid Research, University of Oslo, Oslo, Norway

**Keywords:** Glymphatic system, Meningeal lymphatic vessels, Cerebrospinal fluid, MRI, Neurosurgery

## Abstract

The discovery of the glymphatic system has fundamentally altered our comprehension of cerebrospinal fluid transport and the removal of waste from brain metabolism. In the past decade, since its initial characterization, research on the glymphatic system has surged exponentially. Its potential implications for central nervous system disorders have sparked significant interest in the field of neurosurgery. Nonetheless, ongoing discussions and debates persist regarding the concept of the glymphatic system, and our current understanding largely relies on findings from experimental animal studies. This review aims to address several key inquiries: What methodologies exist for evaluating glymphatic function in humans today? What is the current evidence supporting the existence of a human glymphatic system? Can the glymphatic system be considered distinct from the meningeal-lymphatic system? What is the human evidence for glymphatic-meningeal lymphatic system failure in neurosurgical diseases? Existing literature indicates a paucity of techniques available for assessing glymphatic function in humans. Thus far, intrathecal contrast-enhanced magnetic resonance imaging (MRI) has shown the most promising results and have provided evidence for the presence of a glymphatic system in humans, albeit with limitations. It is, however, essential to recognize the interconnection between the glymphatic and meningeal lymphatic systems, as they operate in tandem. There are some human studies demonstrating deteriorations in glymphatic function associated with neurosurgical disorders, enriching our understanding of their pathophysiology. However, the translation of this knowledge into clinical practice is hindered by the constraints of current glymphatic imaging modalities.

## Introduction

The discovery of the glymphatic system in 2012 [34] sparked a significant shift in our understanding of cerebrospinal fluid (CSF) dynamics and its crucial role in clearing waste from the brain. Over the past few years, there has been a substantial increase in the literature on experimental studies in animals, particularly rodents, with broad implications for the treatment of central nervous system (CNS) diseases (for review see [57]).

The glymphatic system encompasses a brain-wide perivascular transport pathway for fluids and solutes, believed to be pivotal in removing metabolic waste from the brain [52], while also facilitating the transportation of substances to the brain [44]. In rodents, the glymphatic system is by far most active during sleep [79], but its efficacy diminishes with aging [41] and in systemic diseases such as experimental arterial hypertension [48] and diabetes [36]. Moreover, impaired glymphatic function may associate with the accumulation of toxic waste, including amyloid-β, tau, and α-synuclein in the brain, suggesting a significant role in dementia diseases like Alzheimer’s and Parkinson’s [7, 52]. It has also been proposed to play a crucial role in brain edemas resulting from stroke [49] and traumatic brain injury [33].

The glymphatic system has garnered attention in neurosurgical literature, with expectations regarding its potential impact on neurosurgical practices [2, 72]. However, critics highlight unresolved and debated aspects of the glymphatic concept [30, 50]. It is worth noting that the bulk of research on the glymphatic system has been conducted in animals, leaving questions unanswered regarding its translation to humans. From a clinical standpoint, the relevance of the glymphatic system depends on our ability to measure its function or dysfunction, as well as to identify changes in glymphatic function in response to interventions.

Against this backdrop, this review critically examines the following questions: (1) What methodologies exist for evaluating glymphatic function in humans today? (2) What is the current evidence supporting the existence of a human glymphatic system? (3) Can the glymphatic system be considered distinct from the meningeal-lymphatic system? (4) What is the human evidence for glymphatic-meningeal lymphatic system failure in neurosurgical diseases?

### What methodologies exist for evaluating glymphatic function in humans today?

Today, the methods for assessing glymphatic function in humans predominantly hinge on magnetic resonance imaging (MRI) [40, 70]. More modalities are available in animals but are not commented on here. Table 1 provides an overview of the currently used human methods, each with its own set of advantages and disadvantages.

**Table 1:** Main available MRI methods to assess human glymphatic function

MRI Methodology	Advantage	Disadvantage
Intrathecal-contrast-enhanced MRI	Current gold-standard for assessing tracer movement in human brain	Requires spinal puncture. Intrathecal MRI contrast agent used off-label
Intravenous contrast-enhanced MRI	No spinal puncture required	Challenge in defining the glymphatic versus vascular tracer enrichment.
Diffusion tensor image analysis along the perivascular space (DTI-ALPS)	Non-invasive	Assess events within a small region of deep white matter, not necessarily representative for glymphatic function.
White matter perivascular spaces (PVS)	No need for contrast agents.	Limited association between cortical PVS and the PVS of white matter. Heterogeneity of white matter PVS with unknown communication towards subarachnoid CSF
Magnetic resonance encephalography (MREG)	Non-invasive method	Experimental approach lacking established association with glymphatic function.

#### Intrathecal contrast-enhanced MRI

The initial demonstration occurred in a patient investigated for potential CSF leakage, where intrathecally administered gadobutrol (Gadovist, Bayer, GE) enriched brain tissue [15], indicating the free passage of the contrast agent from the subarachnoid space to the cerebral cortex and subcortical white matter. Subsequently, it was revealed that intrathecal gadobutrol enriches the entire brain in a centripetal manner, moving from the cortical surface inward [62, 63, 77]. The extent of tracer enrichment heavily relies on the amount of tracer in the subarachnoid CSF. Drawbacks include the requirement for spinal puncture and the off-label use of gadobutrol for intrathecal administration, which raises concerns about potential toxic effects and brain deposition. However, these concerns may be overstated for several reasons: (a) Toxic effects have not been observed in hundreds of patients receiving intrathecal gadobutrol in doses of 0.25 to 0.50 mmol [12, 28, 69]. (b) Gadobutrol retention in the human brain was not evident after four weeks [64]. (c) Considering that the on-label dosage of intravenous gadobutrol is significantly higher than the intrathecal dosage, CSF concentrations are comparable following intrathecal and intravenous injections [74].

#### Intravenous contrast-enhanced MRI

Due to the necessity of spinal puncture in intrathecal contrast-enhanced MRI, researchers have explored the visualization of glymphatic transport using intravenous contrast agents [81]. The concept is that some contrast enters the CSF, allowing for the evaluation of extravascular transport. However, a major drawback is the difficulty in distinguishing between glymphatic and vascular tracer enrichment since contrast may also leak from blood through the blood-brain-barrier.

#### MRI diffusion tensor image analysis along the perivascular space (DTI-ALPS)

A widely used non-invasive MRI method for glymphatic visualization is diffusion MRI, particularly the DTI-ALPS technique [71]. Despite its increasing popularity, this method has significant limitations [58]: (a) It measures water diffusivity in deep white matter, whereas glymphatic function pertains to solute and fluid transport rather than water transport alone. (b) Events in deep white matter may offer limited insight into glymphatic function, which is primarily a cortical phenomenon. (c) The vasculature in deep white matter and cerebral cortex differs. (d) The perivascular spaces encompass less than 1% of the brain volume [4], and the DTI-ALPS region of interest may not isolate water motion in the perivascular space from other directional water transport in white matter, for instance along axons. Consequently, there are substantial concerns regarding the use of DTI-ALPS as a measure of glymphatic function.

#### Perivascular spaces (PVS) of deep white matter

Another imaging option involves assessing enlarged white matter PVSs as non-invasive measure of glymphatic function [76]. The burden of enlarged PVS in the centrum semiovale and basal ganglia have been proposed as potential non-invasive measures of glymphatic function [53]. However, concerns remain regarding the communication between white matter PVS and CSF, as well as the relationship between events in white matter and the cerebral cortex. There may also exist other confounding factors behind enlarged PVS rather than impaired glymphatic function.

#### Magnetic resonance encephalography (MREG)

Another non-invasive approach to evaluate glymphatic function is ultra-fast MREG [39], which non-invasively assesses three types of physiological measures affecting brain pulsations (cardiac, respiratory and slow waves). While being a promising non-invasive technique, providing for unique insights into brain pulsations, the primary challenge lies in determining the extent to which alterations observed relate to changes in glymphatic function.

Overall, there is currently a scarcity of methods for clinically assessing glymphatic function in humans. Presently, intrathecal contrast-enhanced MRI, as developed by the author and colleagues, is by several considered the gold standard for glymphatic imaging in humans [74].

### What is the current evidence supporting the existence of a human glymphatic system?

**Fig. 1:**
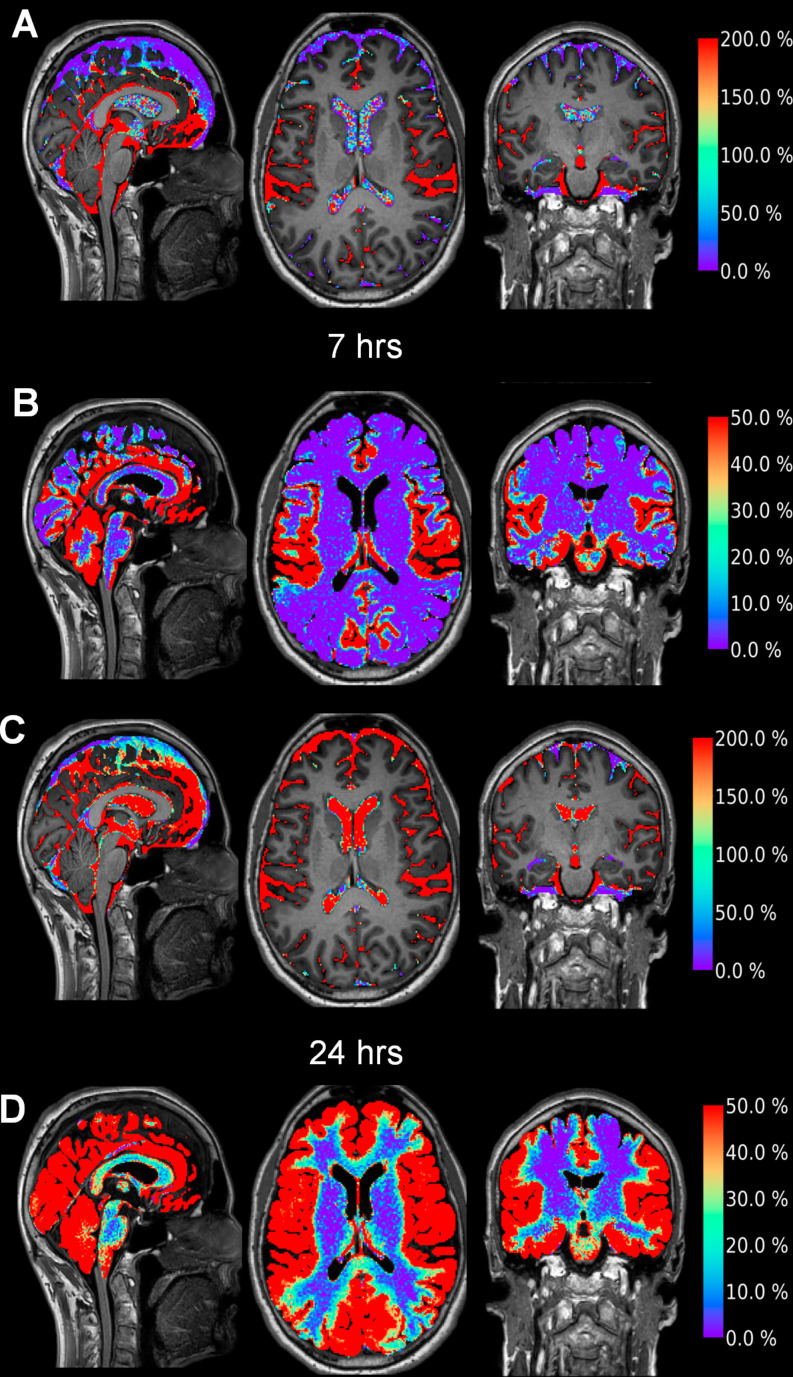
Glymphatic enrichment of a CSF tracer in a human subject is depicted in this figure. Currently, intrathecal contrast-enhanced MRI is considered the gold standard for glymphatic imaging in humans. Following the intrathecal injection of a CSF tracer, such as gadobutrol (Gadovist, Bayer, GE; 0.50 mmol, total volume 1 ml), tracer enrichment is visualized using standardized MRI T1 acquisitions, as previously described. The tracer first enriches the subarachnoid spaces (**A**), then progresses to the cerebral cortex and subcortical white matter (**B**), as indicated by the percentage increase on the color bars to the right at 7 h. It’s worth noting that the strongest enrichment within the subarachnoid space (A) corresponds to the area of strongest enrichment in the cerebral cortex (**B**). By 24 h, tracer enrichment remained comparable in the subarachnoid space but increased in the cerebral ventricles (**C**), while glymphatic enrichment became brain-wide at this time (**D**; percentage increase in tracer enrichment shown on the color bars to the right). The tracer gadobutrol is hydrophilic and does not pass the blood-brain barrier; instead, it remains confined to the extravascular compartment when administered intrathecally. It is a neutral compound with a molecular weight of 604 Da. Images provided by Lars Magnus Valnes, PhD, Department of Neurosurgery, Oslo University Hospital-Rikshospitalet

The current evidence supporting the existence of a human glymphatic system heavily relies on observations made through intrathecal contrast-enhanced MRI, where the contrast agent acts as a CSF tracer. Some principal lines of evidence are depicted in Fig. 1 and can be summarized as follows:

#### Brain-wide distribution of a CSF tracer

**Fig. 2:**
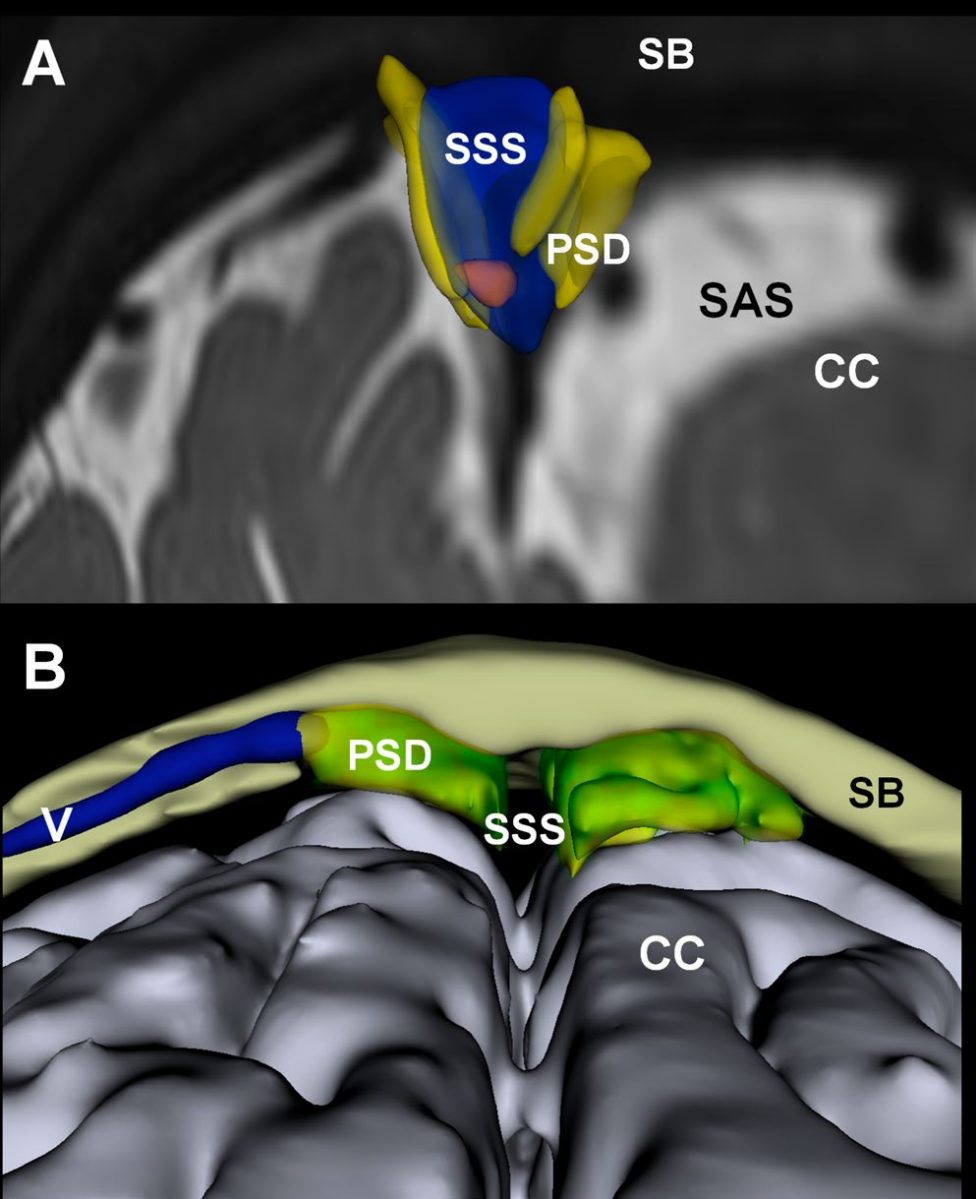
The parasagittal dura. An MRI contrast agent utilized as a cerebrospinal fluid (CSF) tracer enriches the CSF within the subarachnoid space (SAS) and traverses the arachnoid membrane into the dura mater along the superior sagittal sinus (SSS), known as the parasagittal dura (PSD). This phenomenon is depicted here through 3D images generated from T2-FLAIR images, co-registered with brain segmentation and CSF tracer enhancement from T1 GRE at 48 h post-intrathecal tracer injection. (**A**) The superior sagittal sinus (SSS) is highlighted in blue, while the parasagittal dura (PSD) is depicted in yellow. (**B**) The PSD may extend into the marrow of the skull bone (SB). Additionally, a vein (V) within the diploic space is shown. CC: Cerebral cortex. Images by Tomas Sakinis, MD, Department of Radiology, Oslo University Hospital-Rikshospitalet.

The glymphatic system functions as a brain-wide perivascular transport network for fluids and solutes, featuring periarterial influx and perivenous outflux pathways. The contrast agent, acting as a CSF tracer, initially enriches the subarachnoid CSF space and subsequently permeates the entire brain in a centripetal manner, from the cortical surface to subcortical regions [63]. Given the typical 1 mm resolution of MRI, the precise route of tracer passage - whether perivascular along arteries or veins, along the basement membrane of capillaries, or across the pia mater into the interstitial tissue - remains undetermined. Evidence supporting periarterial tracer passage along vessels includes tracer enrichment in the cerebral cortex adjacent to major artery trunks of the subarachnoid space, such as the anterior cerebral artery, middle cerebral artery, and posterior cerebral artery [17, 63]. Importantly, studies administering a CSF tracer to the subarachnoid space of pigs yielded comparable tracer distribution patterns as observed in humans, with immunohistochemistry and microscopic examinations confirming tracer confinement to the perivascular spaces [5]. A notable observation from human studies is that significant, inter-individual enrichment patterns in brain exist [63] (see Fig. 2).

#### Tracer distribution in the human brain compared with the pattern of toxic metabolite accumulation in dementia diseases

The glymphatic system is hypothesized to serve as a clearance pathway for toxic metabolites like amyloid-β, tau in Alzheimer’s disease, and α-synuclein in Parkinson’s disease. The pathological aggregation of these metabolites in dementia diseases follows a characteristic pattern, which aligns to some degree with the distribution of tracer observed in human studies [52].

#### Tracer transport not solely explained by diffusion

The glymphatic concept suggests that perivascular solute transport relies on convective forces (i.e., pressure-gradient forces), with diffusion potentially more prominent in the interstitial tissue. In humans, brain-wide tracer transport occurs over hours [63], a prolonged phenomenon not solely explained by diffusion, indicating the involvement of additional forces [73, 75].

#### Facilitated solute transport along subarachnoid perivascular subarachnoid spaces (PVSAS)

Subpial periarterial influx of CSF constitutes a crucial aspect of the glymphatic system [34]. In humans, facilitated tracer transport occurs along major cerebral vessels anterior cerebral artery, middle cerebral artery and posterior cerebral artery within the subarachnoid space, followed by enrichment of the cerebral cortex where arteries penetrate the brain [17]. This facilitates the antegrade transport of fresh CSF along arteries towards the brain within the PVSAS, while CSF containing waste products empties perivenously into the subarachnoid space outside PVSAS, to be expelled from the subarachnoid space at arachnoid cuff exit (ACE) points [68].

#### CSF tracer dynamics from the human brain is sleep-dependent

In mice, the glymphatic system primarily operates during sleep [79], whereas in humans, clearance of tracer from the cerebral cortex and subcortical white matter significantly decreases after one night of total sleep deprivation [20], albeit to a lesser extent than observed in animals. One night of sleep deprivation also increased accumulation of amyloid-β in the hippocampus and thalamus of healthy volunteers [67]. Modeling studies also suggest a weaker effect of sleep deprivation on tracer clearance in humans than rodents, though the impact in humans remains demonstrable [75]. Furthermore, in patients with chronic impaired sleep quality, tracer enrichment and clearance in the human brain become altered [23].

#### Association between markers of glymphatic function and plasma biomarkers of dementia

The glymphatic system’s primary function is proposed to be the clearance of toxic waste products from brain metabolism, with impaired glymphatic clearance hypothesized to underlie the abnormal aggregation of toxic waste seen in dementia diseases. In humans, markers of glymphatic function derived from CSF tracer assessments correlate with plasma biomarkers of neurodegeneration [24].

### Role of the water channel aquaporin-4 (AQP4) for glymphatic transport in humans

Indirect evidence suggests a potential role of AQP4 in glymphatic transport in humans. Cortical biopsies from patients with idiopathic normal pressure hydrocephalus (iNPH) demonstrate loss of perivascular AQP4 [13, 29]. The iNPH patients also show impaired glymphatic enrichment [63]. However, further investigation is required to determine whether the loss of perivascular AQP4 is a causative mechanism behind the glymphatic failure. In this regard, it is worth noting that a recent study found that acute treatment with the AQP4 inhibitor AER-271 inhibited glymphatic flow in mice, without altering the localization of AQP4 to astrocytic endfeet [26].

#### Evidence of impaired glymphatic clearance in patients

Evidence for a human glymphatic system also relies on the in vivo evidence for impaired glymphatic clearance in patients with iNPH [16, 63] and idiopathic intracranial hypertension (IIH) [21].

In summary, the evidence supporting the existence and function of the human glymphatic system is multi-faceted, encompassing various lines of inquiry and observations.

### Can the glymphatic system be considered distinct from the meningeal-lymphatic system?

In addition to the discovery of the glymphatic system, the rediscovery of meningeal lymphatic vessels capable of draining CSF to dural and extra-dural lymphatic structures represented a breakthrough [3, 45]. The meningeal lymphatic pathways may serve as a final common pathway for the clearance of substances from both the glymphatic pathways and the CSF; its function impairs with age [65]. While the glymphatic system is a brain-wide clearance system involving the CSF, current understanding indicates that clearance primarily occurs to the subarachnoid CSF. The subsequent step involves clearance from the CSF, a process not fully explained by the glymphatic system. Obstruction of this clearance route may exacerbate waste accumulation (including amyloid-β, tau and α-synuclein) and dementia disease progression [9, 10, 54]. Therefore, the glymphatic system should not be viewed in isolation from the meningeal lymphatic system. Additionally, the meningeal lymphatic system plays a crucial role in CNS immunosurveillance, which may significantly impact the glymphatic system. In this regard, it is worth noting that perivascular macrophages play an important role in clearing the perivascular pathways [11].

In human tracer studies, it was observed that tracer in the subarachnoid CSF passed directly to the parasagittal dura (Fig. 2) through the arachnoid membrane (although the exact site of transport was not defined) [59], the marrow of skull bone [60], and even to extracranial lymph nodes [18]. A significant observation is that the amount of tracer in the subarachnoid CSF determines the extent of tracer enrichment in the parasagittal dura as well as in the brain [59, 62]. Therefore, the CSF in the subarachnoid space seems to serve as a reservoir for metabolites, from which substances are transported via lymphatic dural vessels to peripheral lymph nodes and blood.

However, there has been controversy regarding how substances within the subarachnoid CSF are transported to the dura mater, considering the barrier properties of the arachnoid barrier cell layer [78]. A recent significant discovery was the identification of openings in the arachnoid barrier cell layer where bridging veins pass from the cerebral cortex to the dura mater; these openings were delineated by arachnoid cuffs, creating arachnoid cuff exit (ACE) points in the arachnoid where cells and substances may pass along the perivenous basement membrane toward the dura mater [68]. Passage of cells and substances also occurred from outside to CSF.

Imaging the capacity of meningeal lymphatic clearance can pose challenges [61]. Thus, we propose evaluating from plasma samples the CSF-to-blood clearance of an intrathecal tracer, as a surrogate marker of meningeal lymphatic clearance capacity [22]. Pharmacokinetic modeling allows for determining individual CSF-to-blood clearance capacities, revealing significant inter-individual variability [31]. Just as the dose of intravenous drugs can be tailored based on renal clearance function, as measured by the glomerular filtration rate (GFR), so too can the dose of intrathecal drugs be adjusted based on CSF-to-blood clearance function.

It’s worth noting that the primary route for CSF efflux predominantly takes place at the spinal level. Studies showed that peak plasma levels of CSF tracer [31] are observed several hours prior to the peak enrichment of the tracer in the PSD [59]. Modeling studies have further suggested that approximately two-thirds of the total CSF efflux transpires from the spinal canal [75]. Additionally, CSF efflux at the skull base could also be significant, as previously demonstrated experimentally [1].

In summary, in the context of brain clearance, it may be more useful to consider the glymphatic-meningeal lymphatic system as interconnected entities.

### What is the human evidence for glymphatic-meningeal lymphatic system failure in neurosurgical diseases?

In the neurosurgical community, there is a growing awareness of the potential implications of glymphatic failure for neurosurgical diseases [2, 72]. This relates to burgeoning body of experimental literature suggesting a role of glymphatic dysfunction in conditions such as edema following subarachnoid hemorrhage [8, 25], traumatic brain injury [6, 33, 35], post-stroke edema [49], post-hemicraniectomy features [56], subdural hematoma [43, 66], and primary brain tumors [32, 46].

However, the focus of this review is not on experimental animal studies but rather on the human evidence for glymphatic failure in neurological disorders.

#### Idiopathic normal pressure hydrocephalus (iNPH)

This disease stands out as the most extensively studied condition to date. In iNPH, the perivascular spaces of the subarachnoid space (PVSAS) exhibit dysfunction, characterized by widened PVSAS areas and slowed perivascular tracer transport [17]. This is accompanied with enhanced tracer enrichment in the brain and slowed clearance, likely due to impaired glymphatic transport. Notably, this impairment is evident in the entorhinal cortex [16], a region critical for cognitive function [51], suggesting potential clinical relevance to the cognitive decline observed in iNPH patients. Furthermore, this patient group demonstrates pronounced ventricular tracer enrichment caused by tracer reflux into the ventricles [19, 62, 63]. These findings indicate marked alterations in solute transport within the CSF in iNPH, which may contribute to the accumulation of amyloid-β and tau in the cerebral cortex of these patients [42]. The iNPH disease should be considered a combined neurodegenerative and CSF disease where the shunt surgery mainly addresses the CSF component.

#### Idiopathic intracranial hypertension (IIH)

The IIH patients also exhibit evidence of delayed brain-wide tracer clearance [21]. This is of interest given that IIH patients may present with cognitive impairment [80]. Additionally, this patient group presents with an increased number of enlarged white matter perivascular spaces in the centrum semiovale and basal ganglia [37]. While IIH has traditionally been viewed as a CSF or venous obstruction disease, observations of glymphatic failure suggest a more widespread brain effect, which may be interpreted as consistent with histopathological data [14].

#### Subarachnoid hemorrhage (SAH)

Following SAH, increased number of enlarged white matter perivascular spaces in the centrum semiovale was reported [38], which authors attribute to glymphatic failure. A previous non-human primate study also provided evidence of glymphatic dysfunction after SAH [27].

#### Traumatic brain injury (TBI)

Glymphatic function has to a lesser degree been studied in TBI patients, but recent experimental evidence suggests a crucial role of glymphatic function for brain edema [33]. In patients with traumatic brain injury (TBI), those with poor sleep quality exhibit evidence of enlarged white matter perivascular spaces [53]. Additionally, there was a significant positive correlation between the number and volume of these spaces and the number of previous mild TBIs, the severity of post-concussive symptoms, and post-traumatic balance issues [55].

#### Diseases affecting the spinal cord

While diseases affecting the spinal cord have not yet been extensively explored, there is evidence of strong glymphatic enrichment within the spinal cord [47].

Currently, the human evidence for glymphatic alterations in neurosurgical diseases remains limited. However, it is anticipated that this landscape will evolve with further research.

### Future directions

Studies on glymphatic function in neurosurgical diseases have offered new insights into disease mechanisms, yet the assessment of glymphatic function in neurosurgical practice has been minimally implemented. To the best of our knowledge, the one example is use of intrathecal contrast-enhanced MRI in assessment of iNPH patients in our institution [19]. A clear objective for the future is the incorporation of methods for assessing glymphatic and meningeal lymphatic functions before, during, and after interventions. To effect change in neurosurgical practice, the evaluation of glymphatic function must be integrated into treatments or interventions, possibly even on multiple occasions. However, this currently poses a challenge due to the limited availability of methods.

Another crucial goal should be individualized assessments, considering the significant inter-individual variation observed both in glymphatic tracer enrichment in the human brain [63] and in CSF-to-blood clearance [31].

## Conclusion

The discovery of the glymphatic system has sparked a paradigm shift in our comprehension of the role of CSF in CNS function, with growing recognition of potential implications in neurosurgical diseases. While the bulk of research originates from experimental studies, this review has concentrated on evidence gleaned from human studies. Undoubtedly, there is a dearth of methodologies suitable for studying glymphatic function in humans. Intrathecal contrast-enhanced MRI was initially introduced and remains the most valuable methodology, albeit with limitations. There is an imperative need for overcoming these imaging obstacles. Despite these limitations, several lines of evidence suggest the presence of a human glymphatic system that may falter in neurosurgical diseases. However, to impact neurosurgical practice, clinically available tools are required to assess glymphatic and meningeal lymphatic function.

## Abbreviations

ACE: Arachnoid cuff exits
AQP4: Aquaporin-4
CNS: Central nervous system
CSF: Cerebrospinal fluid
DTI-ALPS: Diffusion tensor image analysis along the perivascular space
GFR: Glomerular filtration rate
iNPH: Idiopathic normal pressure hydrocephalus
IIH: Idiopathic intracranial hypertension
MREG: Magnetic resonance encephalography
MRI: Magnetic resonance imaging
PSD: Parasagittal dura
PVS: Perivascular space
PVSAS: Perivascular subarachnoid space
SAH: Subarachnoid hemorrhage
TBI: Traumatic brain injury
